# Appleton’s Journal

**Published:** 1874-03

**Authors:** 


APPLETON'S JOURNAL.—This is one of the best and largest of our literary exchanges. It is always filled with entertaining, instructing and well selected reading. The stories, illustrations, etc., are unsurpassed. Miss Fisher, of N. C., as "Christian Reid," has written for its pages a novel of very great beauty, power and interest, entitled "A Daughter of Bohemia," which is now one of the chief attractions of Appleton's. Some of the best writers of the country are regular contributors, and the editorial management is not excelled by any similar journal. Price $4.00 per annum. D. Appleton & Co., New York.

